# The Hidden Burden of Equipment Failure in Endoscopy: Uncovered and Improved With Digital Technology

**DOI:** 10.7759/cureus.31664

**Published:** 2022-11-19

**Authors:** Anushri Halai, Zameer Mohamed, Pushpakaran Munuswamy, Ashish Kalraiya

**Affiliations:** 1 Emergency Medicine, Mid and South Essex NHS Foundation Trust, London, GBR; 2 Nutrition, Imperial College London, London, GBR; 3 Gastroenterology, Mid and South Essex NHS Foundation Trust, London, GBR; 4 Strategy and Improvement, Mid and South Essex NHS Foundation Trust, London, GBR

**Keywords:** digital reporting tool, time motion study, cognitive task analysis, efficiency costs, endoscopy

## Abstract

Objective

A growing and ageing population combined with severe disruptions across endoscopy services throughout the United Kingdom (UK) during the recent pandemic has accelerated the backlog of patients awaiting endoscopic procedures. This places increased pressure to improve service efficiencies in an attempt to reduce this growing burden. Moreover, beyond repair costs, the full impact of faulty endoscopes on services is not well documented. This study aimed to outline tasks performed to traditionally report a broken endoscope; measure the impact on staff time, efficiency costs and staff morale; and report outcomes of staff experience and productivity when replacing traditional reporting with a digital reporting tool.

Methods

This study was conducted over six months at three endoscopy units. Cognitive-task analysis (CTA) and a time-motion study (TMS) were performed to process map all traditional tasks when an endoscope breaks, and again after a digital reporting tool was implemented. Two staff surveys were conducted. Data was aggregated to determine the overall impact and model efficiency costs.

Results

With traditional processes, on average one faulty endoscope generated 54 tasks, consuming 8 hours 53 minutes of staff time or £325 in efficiency costs, with 60% of staff reporting a negative effect on morale. In comparison, digital reporting generated 41 tasks, consuming 4 hours 31 minutes of time or £147 in efficiency costs, resulting in £45,468 saved annually. Furthermore, 95% of staff said their morale improved, and environmentally all paper-based processes were removed.

Conclusion

This study demonstrated the immense hidden burden of faulty endoscopes. Given the current challenges to endoscopy recovery, digital reporting tools may present an attractive means to minimise disruption to endoscopy services driven through improved equipment maintenance.

## Introduction

Backlog of care

Demand for endoscopic services in the UK has doubled over the last five years, due to an ageing population, increasing burden of gastrointestinal disease and uptake of national bowel cancer screening [1,2]. In May 2020 the joint advisory group (JAG)/British Society of Gastroenterology (BSG) national survey reported that the impact of the COVID-19 pandemic had resulted in significant delays unanimously across NHS endoscopy service providers [3]. At the height of the pandemic, the weekly average number of endoscopy procedures across the UK had reduced by 95% when compared to the pre-pandemic period. Several factors including reduced capacity and public reluctance to undergo endoscopy may have accounted for this significant drop [4]. Consequently, by January 2021, the estimated backlog of NHS endoscopy cases was over 476,000 [5]. Several strategies have been proposed to help eliminate this backlog including increasing capacity to 130% or delaying cases based on higher haemoglobin levels, but despite these interventions, any recovery is likely to be protracted [6]. 

Endoscopy services and equipment failure

Many units have implemented an endoscopy recovery plan to tackle this ominous backlog. The ‘Getting It Right First Time’ (GIRFT) report advocates that endoscopy units increase capacity and improve efficiencies [1]. This will be challenging as service delivery can be complex, requiring specially trained clinicians, patient preparation, administrative tasks, pathology, plus supply and maintenance of endoscopy equipment. Endoscopes, in particular, are intricate devices used in high-volume, thus are susceptible to damage.

The cost and impact of endoscope maintenance are therefore a key consideration. The majority of NHS Trusts will enter into a maintenance agreement with an endoscope service provider, as individual ad hoc repairs or replacement of endoscopes out of warranty can add a significant financial burden. Technical failure can reduce the number of endoscopes in circulation within a unit. A recent time-motion analysis revealed that the timely availability of endoscopes has been implicated as a direct contributor to daily service delays [7]. Thus, minimising the time an endoscope is out of action may improve efficiency within an endoscopy department.

Despite the potential value of optimised endoscope maintenance in aiding the COVID-19 recovery plan, to the best of our knowledge, no literature has been published to itemise the true, wider implications of broken endoscopes on hospital services, including factors such as the impact on staff time, efficiency costs and staff morale. It can be difficult to reliably measure this wider impact as traditional processes for reporting faulty endoscopes can be labour-intensive, unit-specific and paper-based. Studies have shown replacing such traditional processes with digital tools can lead to better data collection and benefits such as “efficiencies in workflow and improvement in communication” [8].

In this study, we aim to (i) define the standard processes traditionally followed when a broken endoscope gets reported (ii) measure the hidden burden of endoscope failure on services, including staff time, efficiency costs and staff morale (iii) create a model to estimate the total cost of endoscope failures and (iv) report outcomes of staff experiences using a digital reporting tool for equipment failure.

## Materials and methods

Clinical setting

This study was conducted over six months at the endoscopy and decontamination units of three NHS hospitals; Basildon and Thurrock University Hospital, Broomfield Hospital, and Southend University Hospital. Whilst these hospitals are all part of Mid and South Essex NHS Foundation Trust, the endoscopy units largely function independently of each other on an operational level. Faulty endoscopes were reported to the hospitals’ Medical Equipment Management Services (MEMS) team and Olympus Medical UK & Ireland (Olympus), with whom the hospitals each had an endoscope maintenance contract. The study was performed during 9 am to 5 pm day shifts, when most endoscopic procedures occur. Nurses, healthcare assistants, doctors, porters and medical engineering staff were recruited if they were full-time staff and had over six months’ experience in their unit. Staff were observed in all areas including endoscopy rooms, decontamination rooms, corridors and administrative rooms. Observers were recruited if they had a healthcare background, either as a clinician or hospital service manager, to ensure they understood different tasks. 

1. Traditional Reporting: Cognitive Task Analysis and Time-Motion Study

The first two months of the study focused on defining the standard processes that were traditionally followed when a broken endoscope gets reported to MEMS and Olympus. This was achieved via cognitive-task analysis (CTA) and a time-motion study (TMS).

**Figure 1:**
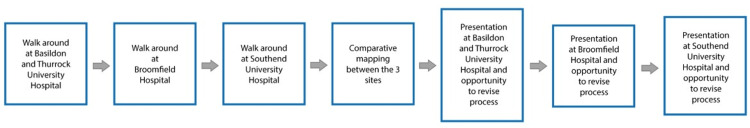
The stepwise process undertaken during Cognitive Task Analysis

CTA was performed with staff at all three sites to process-map the entire sequence of tasks that occur when an endoscope breaks. Staff were led through two cycles of CTA. The first CTA included a walk-around of the units to note every step of the process. During the second CTA, staff were shown the process performed at other departments and they confirmed the accuracy of their initial answers (Figure 1). The CTA identified “routine tasks” that occurred every time an endoscope breaks, such as decontamination, as well as “extra tasks” which occurred ad hoc, such as requesting a loan endoscope.

A TMS was then conducted to measure how long it took staff to complete each of the tasks identified during the CTA. Three observers used a stopwatch to measure the time taken to complete a task on three occurrences, per site. They reported the average time in minutes taken per step, plus the job role of each staff member. “Routine tasks” were measured during clinical practice, whilst “extra tasks” were measured through roleplay due to their ad hoc nature. 

2. Digital Reporting: Cognitive Task Analysis and Time-Motion Study

In the latter four months of the study a digital reporting tool, the MediShout app (MediShout Ltd, London, UK), was used to replace the traditional reporting processes. This digital tool enabled staff to report broken endoscopes directly to MEMS and Olympus, replacing all paper processes and phone calls. Each time staff reported a fault, the app asked them questions to gather prospective, real-time data on the nature of the fault, clinical impact, need for a loan endoscope and impact on morale. Olympus responded to hospital staff via the app to arrange repairs and provide updates. Hospital staff had full visibility of every issue reported and status updates via an online dashboard. CTA and TMS were repeated with the digital process to compare to traditional reporting. 

3. Measuring Staff Satisfaction

Two staff surveys were conducted - one before the implementation of the digital reporting tool and one after the study ended - to further understand the impact on services when an endoscope breaks. Both surveys were undertaken by twenty staff members. The first survey asked about the traditional methods of reporting, and the second about the impact of digital intervention. 

4. Resource Impact Analysis

After all data was gathered from the CTA, TMS, MediShout app, and staff surveys, a resource impact analysis was conducted to estimate the total resource requirement when an endoscope breaks. A model was created first to outline each potential task and assign how much staff time would averagely be consumed per task (see Appendices: Tables 4-6). Next, we calculated the probability of each task occurring based on how frequently the event occurred during the study, except for loan scopes where an average was taken based on the previous twelve months’ activity. Finally, we input which staff were involved with each task, which enabled us to calculate staff time consumed when an endoscope breaks.

To understand the corresponding cost implication of this, each staff member was assigned a cost-per-hour value [9,10]. These costs were applied to the model, based on the staff member’s job role, which enabled us to produce a total expected cost per endoscope failure. The process was completed for both traditional and digital reporting pathways.

## Results

The traditional processes of reporting a broken endoscope

**Figure 2:**
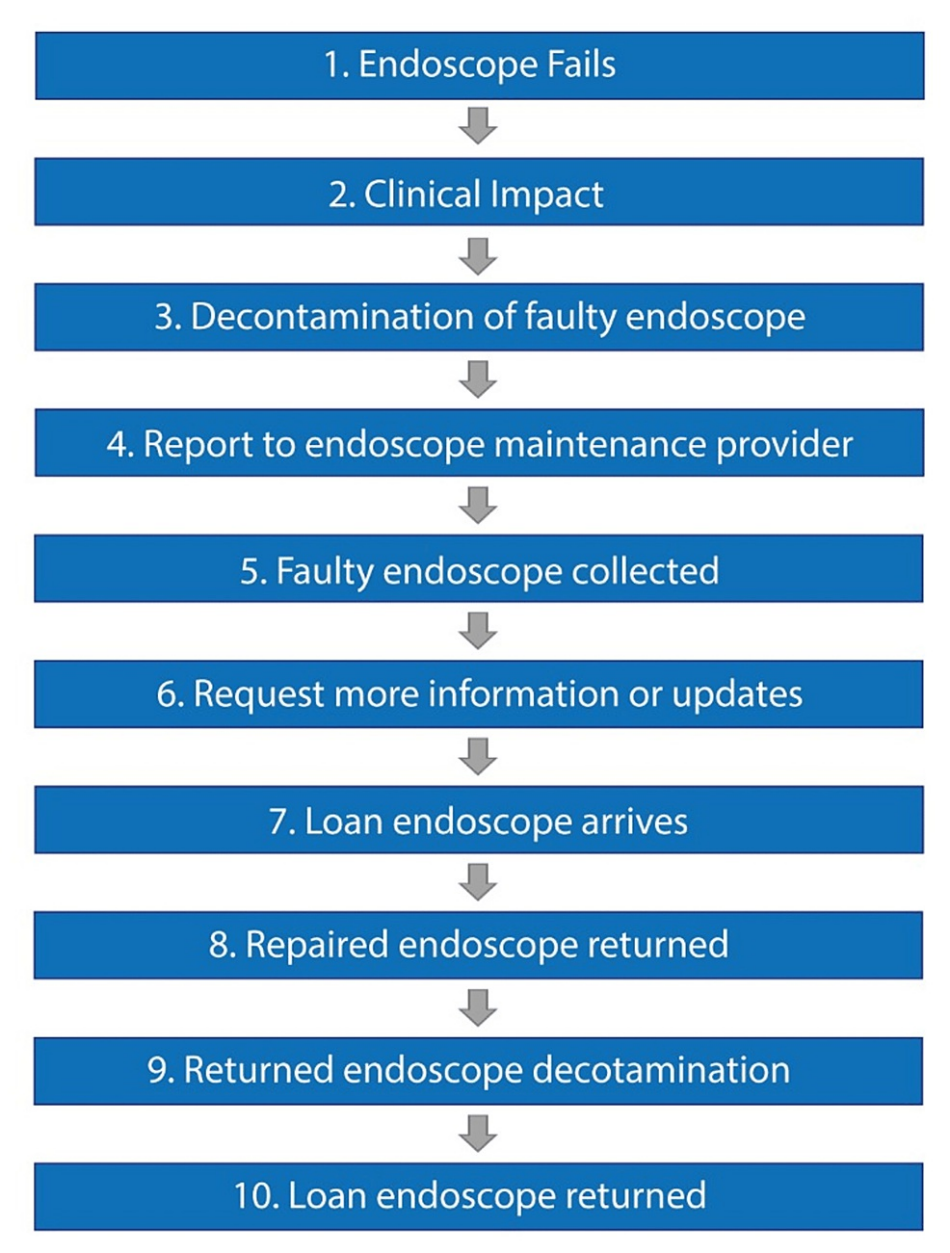
The ten stages of reporting and managing a broken endoscope

Two of the hospitals had identical reporting processes, where faults were reported directly to Olympus. In the third hospital, faulty endoscopes were sent to MEMS, who then reported to Olympus. These differences impacted staff time consumed, such as the time taken to walk from Endoscopy to MEMS. Tasks performed could be grouped into ten main stages (Figure 2). Across these ten stages, reporting directly to Olympus required up to 52 tasks, whereas reporting via MEMS required up to 58 tasks. The combined average across all three sites was 54 tasks (Table 1). 

**Table 1:** The average numbers of tasks, staff time consumed, and efficiency cost from all three endoscopy units – for traditional reporting

Stage	Number of Tasks	Staff Time Consumed (Mins)	Efficiency Cost (£)
1. Endoscope Fails	7	31	25
2. Clinical Impact	1	113	101
3. Decontamination of faulty endoscope	7	62	31
4. Reporting to endoscope maintenance provider	5	53	30
5. Faulty endoscope collected	4	44	18
6. Request more information or updates	3	3	2
7. Loan endoscope arrives	8	60	30
8. Repaired endoscope returns	5	68	34
9. Returned endoscope decontamination	6	60	33
10. Loan endoscope returned	8	41	20
Total	54	533	325

It is important to note that not all the steps in Table 1 and Table 2 occurred every time an endoscope broke. To view all individual tasks that occurred with traditional reporting compared to digital reporting via the MediShout app, in addition to the “probability” of each task occurring, (see Appendices: Tables 7-9). 

**Table 2:** The average numbers of tasks, staff time consumed and efficiency cost, from all three endoscopy units – for digital reporting with the MediShout App

Stage	Number of Tasks	Staff Time Consumed (Mins)	Efficiency Cost (£)
1. Endoscope Fails	7	31	25
2. Clinical Impact	1	10	9
3. Decontamination of faulty endoscope	7	61	32
4. Reporting to endoscope maintenance provider	2	6	3
5. Faulty endoscope collected	4	21	9
6. Request more information or updates	1	5	3
7. Loan endoscope arrives	7	50	24
8. Repaired endoscope returns	1	10	4
9. Returned endoscope decontamination	6	60	31
10. Loan endoscope returned	5	17	8
Total	41	271	147

The hidden burden of endoscope failures on services with traditional processes

Broken endoscopes were found to impact clinical time, efficiency and staff morale. The app revealed each broken endoscope wasted 22.5 minutes of clinical time on average **(**seeAppendices: Tables 4-6**)**. Where staff were reporting directly to Olympus, the total process consumed 8 hours 39 minutes of their time. Where staff reported via MEMS, it consumed 9 hours 23 minutes of their time. Thus, when following traditional processes, one broken endoscope consumed 8 hours 53 minutes of staff time on average. Of note, if the maximum number of tasks is required, staff time consumed can rise to 12 hours 55 mins.

As per Table 3, the pre-implementation survey revealed that 16/20 (80%) staff reported they experienced problems with the endoscopes either weekly (8/20) or monthly (8/20). 18/20 (90%) staff felt an endoscope breaking impacted clinical services, with 7/20 (35%) believing patient care can be impacted. 7/20 (35%) staff reported their endoscope maintenance provider gives feedback when a faulty endoscope is reported. When a fault occurs, 12/20 (60%) staff reported that their morale gets affected. 7/20 (35%) staff perceived that 0-15 minutes of clinical time was wasted by faulty endoscopes. 

**Table 3:** Results of the staff survey performed before the implementation of the MediShout app

Question	Answer
How often do you experience technical problems whilst performing endoscopy	Daily - 2
	Weekly - 8
	Monthly - 8
	More Than Monthly - 2
How much clinical impact do these problems have?	No clinical impact - 2
	Some clinical impact - 16
	Significant clinical impact - 2
If you selected "some clinical impact" or "significant clinical impact": is patient care usually affected?	No - 13
	Yes - 7
Can these issues have effects on staff morale?	No - 8
	Yes - 12
Do you receive feedback when reporting an issue to Olympus?	No – 13
	Yes – 7
On average, how much time is wasted whenever there is a problem?	0-15 mins - 7
	30-6 0mins - 12
	Over 1 hour - 1
Post-Implementation Questionnaire	
Has MediShout improved your communication with your endoscope maintenance provider?	No - 0
	Yes - 19
	N/A - 1
Has MediShout improved the team’s experience of reporting issues to your endoscope maintenance provider?	No - 1
	Yes - 19
	N/A - 0
With MediShout, do you or the team receive feedback after reporting an issue to endoscope maintenance provider?	No - 0
	Yes - 18
	N/A - 2
Has this process change made you more likely to recommend your service provider to colleagues at other hospitals?	No - 0
	Yes - 20
	N/A - 0
Would you prefer to go back to the old ways of writing paper forms and having to make a phone call?	No - 15
	Yes - 4
	N/A - 1
With MediShout has the team’s morale improved?	No - 1
	Yes - 18
	N/A - 1

The model to predict endoscope failures 

The average staff time consumed per faulty endoscope was 8 hours 53 minutes, which equated to £325 in costs, based on the hourly wage of staff involved. Between the 12-month period of April 2019 to March 2020, before the pandemic disrupted service, the NHS Trust sustained 255 endoscope faults. Using the model created, this many faults would annually consume 2,267 hours of staff time, or £82,979 in efficiency costs.

Staff experience with digital reporting 

During the four-month implementation period, the digital reporting tool was used by staff to report 56 faulty endoscopes. Of the users that identified the faulty endoscope, 30/56 (54%) were decontamination staff and 17/56 (30%) were Consultant-level doctors. Of the Consultant-level doctors who identified the fault, 1/17 occurred pre-procedure, 12/17 occurred during procedure and 4/17 occurred post-procedure. Whereas traditional reporting required 54 tasks on average, digital reporting required 41 tasks (Table 2). Staff reported in the survey that the tool saved 20 minutes of clinical time per faulty endoscope, on average. The total amount of staff time consumed per faulty endoscope was 4 hours 31 minutes, equating to £147. Compared to traditional reporting this is a reduction of 4 hours 22 minutes of staff time, resulting in a £178 efficiency saving. Thus, the introduction of this digital innovation could result in an annual saving of 1,115 hours of staff time, or £45,468 for the NHS Trust.

In the post-implementation survey, staff didn’t answer some questions if they weren’t involved in that step of the process. As per Table 3, 19/19 (100%) staff members stated communication improved with their endoscope maintenance provider, with 19/20 (95%) experiencing an improved reporting experience, 18/18 (100%) stating they now received feedback and 20/20 (100%) stating they were now more likely to recommend their services to colleagues. 15/19 (79%) wouldn’t want to return to paper-based, non-digital reporting. 19/20 (95%) of staff believed their morale improved due to the new processes.

## Discussion

COVID-19 has resulted in NHS endoscopy waiting lists rising to almost half a million procedures, making it imperative that endoscopy units perform efficiently and fully utilise existing capacity to clear the backlog. This study provides a revealing insight into the hidden and wide-ranging impact of faulty endoscopes on hospital services, in particular, the impact on staff time, efficiency costs, and staff morale. 

On average, one faulty endoscope generates 54 tasks and consumes 8 hours 53 minutes of staff time, equating to £325 in efficiency costs. This can be as high as 12 hours 55 minutes if all potential tasks are required. Meanwhile, 60% of staff said faulty endoscopes can impact their morale. Contributory factors included the fact that traditional reporting was mainly paper based, with process variation between endoscopy units, and communication between stakeholders often fragmented. For example, whilst 35% of staff reported they didn’t receive feedback from their maintenance provider, it is likely this occurred as staff couldn’t easily communicate updates between themselves or didn’t have full data oversight. 

Staff often underestimated the true burden of tasks generated, despite them being appreciable. For example, 35% of staff perceived only 0-15 minutes of time was wasted when an endoscope breaks whereas our TMS showed reporting alone took 44 minutes. This significant time disparity indicates that staff aren’t aware of all administrative tasks required when an endoscope breaks.

Kramolowsky and colleagues advocated that equipment repair costs alone meant “efforts should be made to minimize instrument breakage” [11]. Considering the additional impact of hidden costs we uncovered, hospitals should seek to proactively prevent the number of endoscope repairs. According to one study, this may be achieved by having endoscopists, nurses and assistants undertake more training in endoscope handling and care to avoid the “nuisance of unwanted and broken endoscopes” [12]. When repair requirements cannot be avoided, then digital technology can minimise the impact by standardising pathways, removing paper-heavy processes, and connecting hospital staff with maintenance providers.

The MediShout app reduced staff time consumed by 4 hours 22 minutes, saving £178 in efficiency costs each time an endoscope broke. This could result in an efficiency saving of £45,468 annually for the NHS Trust. Feedback to staff improved, rising from 35% to 100%, whilst 95% of staff said their morale and reporting experience improved, which aligns with a study that showed “digital technologies also contribute to improving healthcare performance and staff morale if skillfully designed and implemented” [13]. Improving satisfaction with suppliers and departments can bring long-term benefits, with studies showing that better autonomy and communication can help staff retention leading to a better quality of patient care [14]. 

A further motive to incorporate a change in the approach to endoscope maintenance is the potential environmental benefit. Indeed, the spotlight has been recently shone on the high carbon footprint of endoscopy units and has cultivated interest in a more sustainable future endoscopy model. It is conceivable that additional ‘green’ benefits of adopting a technological solution to reduce endoscope faults may reduce paper use, unnecessary decontamination and water use and transit of scopes to and from maintenance providers, all of which are listed as major contributors to endoscopy-related carbon dioxide production in a recent Lancet commentary [15]. Similarly, there has been discussion on the negative environmental impact of disposable endoscopes, with one study estimating that “if all endoscopic procedures were performed with single-use endoscopes and accounting for reprocessing, the net waste mass would increase by 40%” [16]. This further enhances the rationale for improving efficiency within processes that use reusable endoscopes. 

Limitations

Despite providing useful information on current practices in endoscopy maintenance and potential improvements in efficiency, we recognise some limitations to this study. First, there were limitations in the data collection. Endoscopes usually break sporadically and unpredictably, several times per week on average, meaning some tasks had to be role-played and others measured via TMS. Thus, we had to assume that staff enacted role-play accurately. As we couldn’t account for the time taken to switch between tasks or possible distractions to staff during their working day, staff time consumed could be underestimated. In the resource impact analysis, we used the cost of staff time saved, which is a resource efficiency, and not cash-releasing saving. It would be further prudent to understand the basic cost of endoscope repairs for instruments under a maintenance contract. 

Second, although we reported the number of scope failures and tasks required for this reporting, data on the cause of the fault was not recorded. For example, whilst there is a possibility that less experienced clinicians performing endoscopy procedures may contribute to a higher frequency of endoscopy failures, our study did not capture such data. Further focus on these aspects may yield important information that may result in local changes to practice. In addition, although we have clearly documented the economic and efficiency benefits of improving endoscope reporting, we have not recorded the impact of endoscope failure and the potential benefits of integrating a technological solution on procedure numbers performed. This complete information may help to inform a health economic model to measure the true impact of such an intervention.

Finally, though there may be clear buy-in from staff and corresponding improved morale, more quantitative analysis is required to gain a more detailed understanding of staff motivations and perspectives on introducing and sustaining engagement in an electronic reporting platform in this setting. 

## Conclusions

This study demonstrated the immense hidden burden of faulty endoscopes. Each broken endoscope significantly impacts staff time, efficiency, and morale. Given that the backlog of endoscopy care has been compounded by the COVID-19 pandemic, it is imperative that hospitals aim to prevent faulty equipment from becoming a bottleneck in services. The introduction of digital reporting solutions could improve the efficiency in service through a reduction in endoscope maintenance downtime, in addition to having a positive environmental and staff morale impact.

## Appendices

**Table 4:** Summary and comparisons of costs across Mid and South Essex NHS Trust sites

Stage	Basildon/Southend	Broomfield	Average	Basildon/Southend	Broomfield	Average	MediShout	MediShout
	Time Burden (minutes)	Time Burden (minutes)	Time Burden (minutes)	Cost	Cost	Cost	Time Burden (minutes)	Cost
1. Endoscope Fails	30.9	30.9	31	£25.07	£25	£25	31	£25
2. Clinical Impact	112.5	112.5	113	£101.19	£101	£101	10	£9
3. Decontamination of faulty endoscope	61.6	61.6	62	£31.83	£31	£31	61	£32
4. Reporting to endoscope maintenance supplier	44.0	71.0	53	£23.32	£42	£30	6	£3
5. Faulty endoscope collection	47.0	37.0	44	£19.69	£16	£18	21	£9
6. Requestion for more information or updates	3.2	3.2	3	£1.65	£2	£2	5	£3
7. Loan endoscope arrives	57.4	64.2	60	£28.40	£34	£30	50	£24
8. Repaired endoscope returned	66.0	73.0	68	£31.88	£39	£34	10	£4
9. Returned endoscope decontamination	60.0	60.0	60	£31.00	£37	£33	60	£31
10. Loan endoscope returned	36.1	49.5	41	£18.21	£25	£20	17	£8
Total	519	563	533	£312	£352	£325	271	£147

**Table 5:** Comparison of hours and cost savings with MediShout

Task	Time Burden (minutes)	Cost of Time Per Repair	Time Burden (With MediShout) (minutes)	Cost of Time Per Repair (With MediShout)
1. Endoscope Fails	31	£25	31	£25
2. Clinical Impact	113	£101	10	£9
3. Decontamination of faulty endoscope	62	£31	61	£32
4. Reporting to endoscope maintenance supplier	53	£30	6	£3
5. Faulty endoscope collection	44	£18	21	£9
6. Request for more information or updates	3	£2	5	£3
7. Loan endoscope arrives	60	£30	50	£24
8. Repaired endoscope returned	68	£34	10	£4
9. Returned endoscope decontamination	60	£33	60	£31
10. Loan endoscope returned	41	£20	17	£8
Total	535 (8.9 hours)	£325	271 (4.5 hours)	£147

**Table 6:** Comparison of repair hours and costs saved with MediShout

Repairs Per Year	Hours Wasted	Cost of Time Per Repair	Hours Wasted (With MediShout)	Cost of Time Per Repair (With MediShout)
255	2,267	£82,979	1,152	£37,511

**Table 7:** A model to demonstrate tasks, time consumption, and financial costs when using MediShout (digital reporting) MEMS: Medical Equipment Management Services; HCA: health care assistant; EBME: electrical and biomedical engineer

Stage	Tasks	Assessment	Time consumed (minutes)	Probability	Doctors (n)	Nurses (n)	HCAs (n)	EBMEs (n)	Porters (n)	Sum time loss per step (minutes)	Contribution time (minutes)	Sum cost per step	Contrubution cost
1. Endoscope Fails													
Before or During Procedure & new endoscope needed	Identify fault - decision made to use a new scope	Interview	5	14.81%	1	3	1			25	3.7	£22.49	£3.33
Before or During Procedure & new endoscope needed	HCA get new scope from Decontamination clean room	Timed	6	14.81%	1	3	1			30	4.4	£26.99	£4.00
Before or During Procedure & new endoscope needed	HCA/Nurse prep the scope for usage at the bedside	Timed	9	14.81%	1	3	1			45	6.7	£40.48	£5.99
Before or During Procedure but procedure can continue	HCA/Nurse aware that the fault needs to be reported. Time lost due to inspection and attempted self resolve	Interview	6	30.00%	1	3	1			30	9.0	£26.99	£8.10
Post-Procedure	Fault discovered during bedside clean	Timed	5	100.00%			1			5	5.0	£2.58	£2.58
All	Bedside clean performed by endoscopy staff	Interview	2	3.70%			0			0	0.0	£0.00	£0.00
All	Scope brought from procedure room to Decontamination room	Timed	2	100.00%			1			2	2.0	£1.03	£1.03
2. Clinical Impact													
	Clinical time lossed due to impact of scope breaking (median/mean number from App data, hence probability 100%)	Questionnaire	2	100.00%	1	3	1			10	10.0	£9.00	£9.00
3. Decontamination of faulty endoscope	Manual wash of scope in Dirty room	Timed	8	100.00%			1			8	8.0	£4.13	£4.13
	Fault discovered during manual wash	Timed	2	12.96%						0	0.0	£0.00	£0.00
	Endoscope gets scanned in via Health Edge scanner	Timed	2	100.00%			1			2	2.0	£1.03	£1.03
	Scope is loaded into the washer-disinfector	Timed	4	100.00%			1			4	4.0	£2.07	£2.07
	Washer runs a 30 minute wash of the scope	Timed	30	100.00%			1			30	30.0	£15.50	£15.50
	Fault discovered during disinfection process	Interview	29	42.60%			1			29	12.4	£14.98	£6.38
	Endoscope removed from washer-disinfector in clean room	Timed	5	100.00%			1			5	5.0	£2.58	£2.58
4. Reporting to endoscope maintenance provider													
	Fault is reported via the MediShout Web-App	Roleplay	4	100.00%			1			4	4.0	£2.07	£2.07
	Endoscopy staff print the form to be added with the faulty scope	Roleplay	2	100.00%			1			2	2.0	£1.03	£1.03
5. Faulty endoscope collected													
	Scope packaged up with Olympus decontamination form, ready for dispatch	Roleplay	4	100.00%			1			4	4.0	£2.07	£2.07
	Scope collected by porters from Endoscopy, with transit paperwork signed (only broomfield)	Roleplay	4	24.00%			1		1	8	1.9	£3.62	£0.87
	Staff must take scope to Stores then walk back again (only broomfield)	Roleplay	22	24.00%					1	22	5.3	£8.56	£2.05
	Scope collected by Olympus courier and paperwork signed (Stores for Broomfield, endoscopy for Basildon/Southend)	Roleplay	10	100.00%					1	10	10.0	£3.89	£3.89
6. Request more information or updates													
	All communication done automatically via App if needed e.g. asking for more information or updating staff via App chat function	Interview	5	100.00%			1			5	5.0	£2.58	£2.58
7. Loan endoscope arrives													
	Stores receives Loan scope from Olympus courier, signs this in and walks it over to Endoscopy	Roleplay	22	61.11%					1	22	13.4	£8.56	£5.23
	Loan scope inspected by Endoscopy team who scan this in with Health Edge onto their inventory list	Roleplay	13	61.11%			1			13	7.9	£6.72	£4.10
	The endoscopy team perform a manual wash of the loan scope	Timed	8	61.11%			1			8	4.9	£4.13	£2.53
	Endoscopy team load this onto the decontamination machine	Timed	4	61.11%			1			4	2.4	£2.07	£1.26
	The Endoscopy team leaves the loan scope to be decontaminated in the machine	Timed	30	61.11%			1			30	18.3	£15.50	£9.47
	Loan Endoscope removed from washer-disinfector in clean room	Timed	5	61.11%			1			5	3.1	£2.58	£1.58
	Loan scope enters circulation	Timed	0	61.11%						0	0.0		
8. Repaired endoscope returns													
	Olympus courier delivers repaired scope to Endoscopy suite, information automatically updated on MEMS by MediShout	Roleplay	10	100.00%					1	10	10.0	£3.89	£3.89
9. Returned endoscope decontamination													
	Repaired scope unpacked & inspected by Endoscopy team who scan this in with Health Edge onto their inventory list	Roleplay	13	100.00%			1			13	13.0	£6.72	£6.72
	The endoscopy team perform a manual wash of the repaired scope	Timed	8	100.00%			1			8	8.0	£4.13	£4.13
	Endoscopy team load the repaired scope onto the decontamination machine	Timed	4	100.00%			1			4	4.0	£2.07	£2.07
	Washer runs a 30 minute wash of the scope	Timed	30	100.00%			1			30	30.0	£15.50	£15.50
	Endoscope removed from Washer in clean room	Timed	5	100.00%			1			5	5.0	£2.58	£2.58
	Repaired endoscope enters circulation	N/A	0	100.00%						0	0.0		
10. Loan endoscope returned													
	The loan scope is decontaminated before returning to Olympus (timing not counted as usual scopes need decontaminating anyway)	Timed	0	61.11%						0	0.0		
	Scope wash is complete and a ticket from Health Edge is printed, showing Olympus the scope has been through a cleaning process	Roleplay	2	61.11%			1			2	1.2	£1.03	£0.63
	The Endoscopy team complete the Olympus Decontamination form	Roleplay	8	61.11%			1			8	4.9	£4.13	£2.53
	The Endoscopy team puts faulty scope and Olympus decontamination form into Olympus carrier case. Olympus notified Loan scope must be collected	Roleplay	7	61.11%			1			7	4.3	£3.62	£2.21
	Olympus courier collects faulty scope from endoscopy, who must sign out the loan scope on their ledger	Roleplay	10	61.11%					1	10	6.1	£3.89	£2.38

**Table 8:** A model to demonstrate tasks, time consumption, and financial costs at Basildon and Southend hospitals (endoscopy-led reporting) MEMS: Medical Equipment Management Services; HCA: health care assistant; EBME: electrical and biomedical engineer

Stage	Tasks	Assessment	Time (minutes)	Probability	Doctors (n)	Nurses (n)	HCAs (n)	EBMEs (n)	Porters (n)	Sum time loss (minutes)	Contribution time (minutes)	Sum cost per step	Contrubution cost
1. Endoscope Fails													
Before or During Procedure & new endoscope needed	Identify fault - decision made to use a new scope	Interview	5	14.81%	1	3	1			25	3.7	£22.49	£3.33
Before or During Procedure & new endoscope needed	HCA get new scope from Decontamination clean room	Timed	6	14.81%	1	3	1			30	4.4	£26.99	£4.00
Before or During Procedure & new endoscope needed	HCA/Nurse prep the scope for usage at the bedside	Timed	9	14.81%	1	3	1			45	6.7	£40.48	£5.99
Before or During Procedure but procedure can continue	HCA/Nurse aware that the fault needs to be reported. Time lost due to inspection and attempted self resolve	Interview	6	30.00%	1	3	1			30	9.0	£26.99	£8.10
Post-Procedure	Fault discovered during bedside clean	Interview	2	3.70%			1			2	0.1	£1.03	£0.04
All	Bedside clean performed by endoscopy staff	Timed	5	100.00%			1			5	5.0	£2.58	£2.58
All	Scope brought from procedure room to Decontamination room	Timed	2	100.00%			1			2	2.0	£1.03	£1.03
2. Clinical Impact													
	Clinical time lossed due to impact of scope breaking (median/mean number from App data, hence probability 100%)	App	22.5	100.00%	1	3	1			112.5	112.5	£101.19	£101.19
3. Decontamination of faulty endoscope													
	Manual wash of scope in Dirty room	Timed	8	100.00%			1			8	8.0	£4.13	£4.13
	Fault discovered during manual wash	Timed	2	12.96%			1			2	0.3	£1.03	£0.13
	Endoscope gets scanned in via Health Edge scanner	Timed	2	100.00%			1			2	2.0	£1.03	£1.03
	Scope is loaded into the washer-disinfector	Timed	4	100.00%			1			4	4.0	£2.07	£2.07
	Washer runs a 30 minute wash of the scope	Timed	30	100.00%			1			30	30.0	£15.50	£15.50
	Fault discovered during disinfection process	Interview	29	42.60%			1			29	12.4	£14.98	£6.38
	Endoscope removed from washer-disinfector in clean room	Timed	5	100.00%			1			5	5.0	£2.58	£2.58
4. Reporting to endoscope maintenance provider													
	Administrative paperwork for reporting scope is gathered	Roleplay	3	100.00%			1			3	3.0	£1.55	£1.55
	Endoscopy team find the Olympus number, find an available phone, and call Olympus to report the issue. A loan scope can be requested. Call can be missed adding wasted time.	Roleplay	21	100.00%			1			21	21.0	£10.85	£10.85
	Olympus Keymed decontamination form completed	Roleplay	8	100.00%			1			8	8.0	£4.13	£4.13
	Staff complete internal consignment note	Roleplay	2	100.00%			1			2	2.0	£1.03	£1.03
	Staff update MEMS team of the faulty scope	Roleplay	5	100.00%			1	1		10	10.0	£5.76	£5.76
5. Faulty endoscope collected													
	Scope packaged up with Olympus decontamination form, ready for dispatch	Roleplay	7	100.00%			1			7	7.0	£3.62	£3.62
	Scope collected by porters from Endoscopy, with transit paperwork signed	Roleplay	4	100.00%			1		1	8	8.0	£3.62	£3.62
	Staff must take scope to Stores then walk back again	Roleplay	22	100.00%					1	22	22.0	£8.56	£8.56
	Scope collected by Olympus courier, paperwork signed by Stores team	Roleplay	10	100.00%					1	10	10.0	£3.89	£3.89
6. Request more information or updates													
	Olympus call hospital to get more information needed for the repair (successful)	Interview	10	10.00%			1			10	1.0	£5.17	£0.52
	Olympus call hospital to get more information needed for the repair (but the person they need is not avilable)	Interview	2	50.00%			1			2	1.0	£1.03	£0.52
	Hospital staff call Olympus to get progress updates on the repair	Interview	6	20.00%			1			6	1.2	£3.10	£0.62
7. Loan endoscope arrives													
	Stores receives Loan scope from Olympus courier, signs this in and walks it over to Endoscopy	Roleplay	22	61.11%					1	22	13.4	£8.56	£5.23
	Loan scope inspected by Endoscopy team who scan this in with Health Edge onto their inventory list	Roleplay	13	61.11%			1			13	7.9	£6.72	£4.10
	Endoscopy team notify MEMS that Loan Scope has been received	Roleplay	6	61.11%			1	1		12	7.3	£6.91	£4.22
	The endoscopy team perform a manual wash of the loan scope	Timed	8	61.11%			1			8	4.9	£4.13	£2.53
	Endoscopy team load this onto the decontamination machine	Timed	4	61.11%			1			4	2.4	£2.07	£1.26
	The Endoscopy team leaves the loan scope to be decontaminated in the machine	Timed	30	61.11%			1			30	18.3	£15.50	£9.47
	Loan Endoscope removed from washer-disinfector in clean room	Timed	5	61.11%			1			5	3.1	£2.58	£1.58
	Loan scope enters circulation	Timed	0	61.11%									
8. Repaired endoscope returns													
	Olympus call the Endoscopy team to notify them the scope will be delivered back	Interview	10	100.00%			1			10	10.0	£5.17	£5.17
	Olympus courier delivers repaired scope to Stores, who walk this back to the Endoscopy suite	Roleplay	22	100.00%					1	22	22.0	£8.56	£8.56
	Endoscopy team receive repaired scope, update internal logs in clean room, then scan in Olympus paperwork	Roleplay	24	100.00%			1			24	24.0	£12.40	£12.40
	Endoscopy team notify MEMS the repaired scope has returned	Roleplay	5	100.00%			1	1		10	10.0	£5.76	£5.76
9. Returned endoscope decontamination													
	Repaired scope unpacked & inspected by Endoscopy team who scan this in with Health Edge onto their inventory list	Roleplay	13	100.00%			1			13	13.0	£6.72	£6.72
	The endoscopy team perform a manual wash of the repaired scope	Timed	8	100.00%			1			8	8.0	£4.13	£4.13
	Endoscopy team load the repaired scope onto the decontamination machine	Timed	4	100.00%			1			4	4.0	£2.07	£2.07
	Washer runs a 30 minute wash of the scope	Timed	30	100.00%			1			30	30.0	£15.50	£15.50
	Endoscope removed from Washer in clean room	Timed	5	100.00%			1			5	5.0	£2.58	£2.58
	Repaired endoscope enters circulation	N/A	0	100.00%									
10. Loan endoscope returned													
	The loan scope is decontaminated before returning to Olympus (timing not counted as usual scopes need decontaminating anyway)	Timed	0	61.11%									
	Scope wash is complete and a ticket from Health Edge is printed, showing Olympus the scope has been through a cleaning process	Roleplay	2	61.11%			1			2	1.2	£1.03	£0.63
	The Endoscopy team complete the Olympus Decontamination form	Roleplay	8	61.11%			1			8	4.9	£4.13	£2.53
	The Endoscopy team puts faulty scope and Olympus decontamination form into Olympus carrier case. Olympus notified Loan scope must be collected	Roleplay	7	61.11%			1			7	4.3	£3.62	£2.21
	The Endoscopy team take the packaged scope and walk this down to Stores, sign a ledger then walk back to the Endoscopy suite	Roleplay	22	61.11%			1			22	13.4	£11.37	£6.95
	Olympus courier collects faulty scope from Stores, who must sign out the loan scope on their ledger	Roleplay	10	61.11%					1	10	6.1	£3.89	£2.38
	Endoscopy team notify MEMS than Loan Scope has been returned	Roleplay	5	61.11%			1	1		10	6.1	£5.76	£3.52

**Table 9:** A model to demonstrate tasks, time consumption, and financial costs at Broomfield hospital (MEMS-led reporting) MEMS: Medical Equipment Management Services; HCA: health care assistant; EBME: electrical and biomedical engineer

Stage	Tasks	Assessment	Time (minutes)	Probability	Doctors (n)	Nurses (n)	HCAs (n)	EBMEs (n)	Porters (n)	Sum time loss (minutes)	Contribution time (minutes)	Sum cost per step	Contribution cost
1. Endoscope Fails													
Before or During Procedure & new endoscope needed	Identify fault - decision made to use a new scope	Interview	5	14.81%	1	3	1			25	3.7	£22.49	£3.33
Before or During Procedure & new endoscope needed	HCA get new scope from Decontamination clean room	Timed	6	14.81%	1	3	1			30	4.4	£26.99	£4.00
Before or During Procedure & new endoscope needed	HCA/Nurse prep the scope for usage at the bedside	Timed	9	14.81%	1	3	1			45	6.7	£40.48	£5.99
Before or During Procedure but procedure can continue	HCA/Nurse aware that the fault needs to be reported. Time lost due to inspection and attempted self resolve	Interview	6	30.00%	1	3	1			30	9.0	£26.99	£8.10
Post-Procedure	Fault discovered during bedside clean	Timed	5	100.00%			1			5	5.0	£2.58	£2.58
All	Bedside clean performed by endoscopy staff	N/A	2	3.70%			1			2	0.1	£1.03	£0.04
All	Scope brought from procedure room to Decontamination room	Timed	2	100.00%			1			2	2.0	£1.03	£1.03
2. Clinical Impact													
	Clinical time lossed due to impact of scope breaking (median/mean number from App data, hence probability 100%)	App	22.5	100.00%	1	3	1			112.5	112.5	£101.19	£101.19
3. Decontamination of faulty endoscope													
	Manual wash of scope in Dirty room	Timed	8	100.00%			1			8	8.0	£4.13	£4.13
	Fault discovered during manual wash	Timed	2	12.96%			1			2	0.3	£1.03	£0.13
	Endoscope gets scanned in via Health Edge scanner	Timed	2	100.00%			1			2	2.0	£1.03	£1.03
	Scope is loaded into the washer-disinfector	Timed	4	100.00%			1			4	4.0	£2.07	£2.07
	Washer runs a 30 minute wash of the scope	Timed	30	100.00%			1			30	30.0	£15.50	£15.50
	Fault discovered during disinfection process	Interview	29	42.60%			1			29	12.4	12.4	5.262804
	Endoscope removed from washer-disinfector in clean room	Timed	5	100.00%			1			5	5.0	£2.58	£2.58
4. Reporting to endoscope maintenance provider													
	Endoscopy staff calls MEMS to flag that there is a faulty scope to be sent out for repair	Roleplay	5	100.00%			1	1		10	10.0	£5.76	£5.76
	The Endoscopy team or porter take the scope down to the MEMS office	Roleplay	20	100.00%			1			20	20.0	£10.33	£10.33
	MEMS admin team recieves the scope and logs the issue onto Medusa	Roleplay	9	100.00%				1		9	9.0	£5.71	£5.71
	Staff call Olympus to report the issue, calls can be missed	Roleplay	21	100.00%				1		21	21.0	£13.33	£13.33
	MEMS team member updates Medusa with the job reference number and relevant details and prints decontamination details from Medusa	Roleplay	6	100.00%				1		6	6.0	£3.81	£3.81
	MEMS complete the Olympus Keymed decontamination form via Medusa (different to the other hospitals)	Roleplay	5	100.00%				1		5	5.0	£3.17	£3.17
5. Faulty endoscope collected													
	Scope packaged up with Olympus decontamination form, ready for dispatch	Roleplay	7	100.00%				1		7	7.0	£4.44	£4.44
	Scope collected from MEMS, with transit paperwork signed	Roleplay	4	100.00%					1	4	4.0	£1.56	£1.56
	Staff must take scope to Stores then walk back again	Roleplay	16	100.00%					1	16	16.0	£6.22	£6.22
	Scope collected by Olympus courier, paperwork signed by Stores team	Roleplay	10	100.00%					1	10	10.0	£3.89	£3.89
6. Request more information or updates													
	Olympus call hospital to get more information needed for the repair (successful)	Interview	10	10.00%			1			10	1.0	£5.17	£0.52
	Olympus call hospital to get more information needed for the repair (missed call)	Interview	2	50.00%			1			2	1.0	£1.03	£0.52
	Hospital staff call Olympus to get progress updates on the repair	Interview	6	20.00%			1			6	1.2	£3.10	£0.62
7. Loan endoscope arrives													
	Stores receives Loan scope from Olympus courier, signs this in and walks it over to MEMS	Roleplay	16	61.11%					1	16	9.8	£6.22	£3.80
	MEMS update the information on Medusa	Roleplay	7	61.11%				1		7	4.3	£4.44	£2.71
	MEMS drop the loan scope to Endoscopy and walk back	Roleplay	20	61.11%				1		20	12.2	£12.69	£7.76
	Loan scope inspected by Endoscopy team who scan this in with Health Edge onto their inventory list	Roleplay	13	61.11%			1			13	7.9	£6.72	£4.10
	The endoscopy team perform a manual wash of the loan scope	Timed	8	61.11%			1			8	4.9	£4.13	£2.53
	Endoscopy team load this onto the decontamination machine	Timed	4	61.11%			1			4	2.4	£2.07	£1.26
	The Endoscopy team leaves the loan scope to be decontaminated in the machine	Timed	30	61.11%			1			30	18.3	£15.50	£9.47
	Endoscope removed from washer-disinfector in clean room	Timed	5	61.11%			1			5	3.1	£2.58	£1.58
	When the repaired scope is returned, the endoscopy team then know to return the loan scope. Endoscopy team check which loan scope needs to be returned.	Roleplay	2	61.11%			1			2	1.2	£1.03	£0.63
8. Repaired endoscope returns													
	Olympus call MEMS to notify them the scope will be delivered back.	Interview	10	100.00%				1		10	10.0	£6.35	£6.35
	Olympus courier delivers repaired scope to Stores, who do paperwork	Roleplay	10	100.00%					1	10	10.0	£3.89	£3.89
	Stores walk this back to MEMS and walk back	Roleplay	16	100.00%					1	16	16.0	£6.22	£6.22
	MEMS update the information on Medusa	Roleplay	7	100.00%				1		7	7.0	£4.44	£4.44
	MEMS drop the repaired scope to Endoscopy	Roleplay	20	100.00%				1		20	20.0	£12.69	£12.69
	Endoscopy team receive repaired scope, update internal paperwork logistics logs	Roleplay	10	100.00%			1			10	10.0	£5.17	£5.17
9. Returned endoscope decontamination													
	Repaired scope unpacked & inspected by Endoscopy team who scan this in with Health Edge onto their inventory list	Roleplay	13	100.00%			1			13	13.0	13.0	£13.00
	The endoscopy team perform a manual wash of the repaired scope	Timed	8	100.00%			1			8	8.0	£4.13	£4.13
	Endoscopy team load the repaired scope onto the decontamination machine	Timed	4	100.00%			1			4	4.0	£2.07	£2.07
	Washer runs a 30 minute wash of the scope	Timed	30	100.00%			1			30	30.0	£15.50	£15.50
	Endoscope removed from Washer in clean room	Timed	5	100.00%			1			5	5.0	£2.58	£2.58
	Loan endoscope enters circulation	N/A	0										
10. Loan endoscope returned													
	The loan scope is decontaminated before returning to Olympus (timing not counted as usual scopes need decontaminating anyway)	N/A	0	61.11%						0	0.0		
	Scope wash is complete and a ticket from Health Edge is printed, showing Olympus the scope has been through a cleaning process	Roleplay	2	61.11%			1			2	1.2	1.2	£0.75
	The Endoscopy team complete the Olympus Decontamination form	Roleplay	8	61.11%			1			8	4.9	4.9	£2.99
	Endoscopy staff calls MEMS to flag that the loan scope is ready to be sent back	Roleplay	5	61.11%			1	1		10	6.1	£5.76	£3.52
	The Endoscopy team or porter take the scope down to the MEMS office	Roleplay	20	61.11%			1			20	12.2	£10.33	£6.31
	MEMS admin team update Medusa	Roleplay	7	61.11%				1		7	4.3	£4.44	£2.71
	Scope collected from MEMS- transit paperwork need to be signed	Roleplay	4	61.11%				1	1	8	4.9	£4.09	£2.50
	Staff travel time taking scope to stores + return journey	Roleplay	16	61.11%					1	16	9.8	£6.22	£3.80
	Scope collected by Olympus staff +signs for collection	Roleplay	10	61.11%					1	10	6.1	£3.89	£2.38
